# Isotopic insights into microbial sulfur cycling in oil reservoirs

**DOI:** 10.3389/fmicb.2014.00480

**Published:** 2014-09-19

**Authors:** Christopher G. Hubbard, Yiwei Cheng, Anna Engelbrekston, Jennifer L. Druhan, Li Li, Jonathan B. Ajo-Franklin, John D. Coates, Mark E. Conrad

**Affiliations:** ^1^Earth Sciences Division, Lawrence Berkeley National LaboratoryBerkeley, CA, USA; ^2^Department of Plant and Microbial Biology, University of California at BerkeleyBerkeley, CA, USA; ^3^Department of Geological and Environmental Sciences, Stanford UniversityStanford, CA, USA; ^4^Department of Energy and Mineral Engineering, Pennsylvania State UniversityUniversity Park, PA, USA

**Keywords:** microbial sulfate reduction, stable isotopes, souring, reactive transport modeling, reservoir modeling, oil reservoirs

## Abstract

Microbial sulfate reduction in oil reservoirs (biosouring) is often associated with secondary oil production where seawater containing high sulfate concentrations (~28 mM) is injected into a reservoir to maintain pressure and displace oil. The sulfide generated from biosouring can cause corrosion of infrastructure, health exposure risks, and higher production costs. Isotope monitoring is a promising approach for understanding microbial sulfur cycling in reservoirs, enabling early detection of biosouring, and understanding the impact of souring. Microbial sulfate reduction is known to result in large shifts in the sulfur and oxygen isotope compositions of the residual sulfate, which can be distinguished from other processes that may be occurring in oil reservoirs, such as precipitation of sulfate and sulfide minerals. Key to the success of this method is using the appropriate isotopic fractionation factors for the conditions and processes being monitored. For a set of batch incubation experiments using a mixed microbial culture with crude oil as the electron donor, we measured a sulfur fractionation factor for sulfate reduction of −30‰. We have incorporated this result into a simplified 1D reservoir reactive transport model to highlight how isotopes can help discriminate between biotic and abiotic processes affecting sulfate and sulfide concentrations. Modeling results suggest that monitoring sulfate isotopes can provide an early indication of souring for reservoirs with reactive iron minerals that can remove the produced sulfide, especially when sulfate reduction occurs in the mixing zone between formation waters (FW) containing elevated concentrations of volatile fatty acids (VFAs) and injection water (IW) containing elevated sulfate. In addition, we examine the role of reservoir thermal, geochemical, hydrological, operational and microbiological conditions in determining microbial souring dynamics and hence the anticipated isotopic signatures.

## Introduction

Microbial reduction of sulfate to sulfide (also known as microbial “souring” or “biosouring” by the oil and gas industry) is arguably the most deleterious microbial process that oil operators face during oil production (Youssef et al., 2009; Gieg et al., 2011). The sulfide formed presents health risks to workers when present in the gas phase as hydrogen sulfide (H_2_S) and needs to be removed from the crude oil before it can be refined, resulting in a more expensive end-product. Sulfide is also highly corrosive, requiring significant investment in corrosion-resistant infrastructure, either before production occurs or as an expensive retrofit involving periods of non-production. A suite of tools are therefore necessary to help understand, predict, prevent and mitigate microbial souring. Here we argue that stable isotopes are currently under-utilized as a microbial souring tool and illustrate how they may potentially be used to provide an early indication of souring and gain insight into reservoir sulfur cycling.

**Figure 1:**
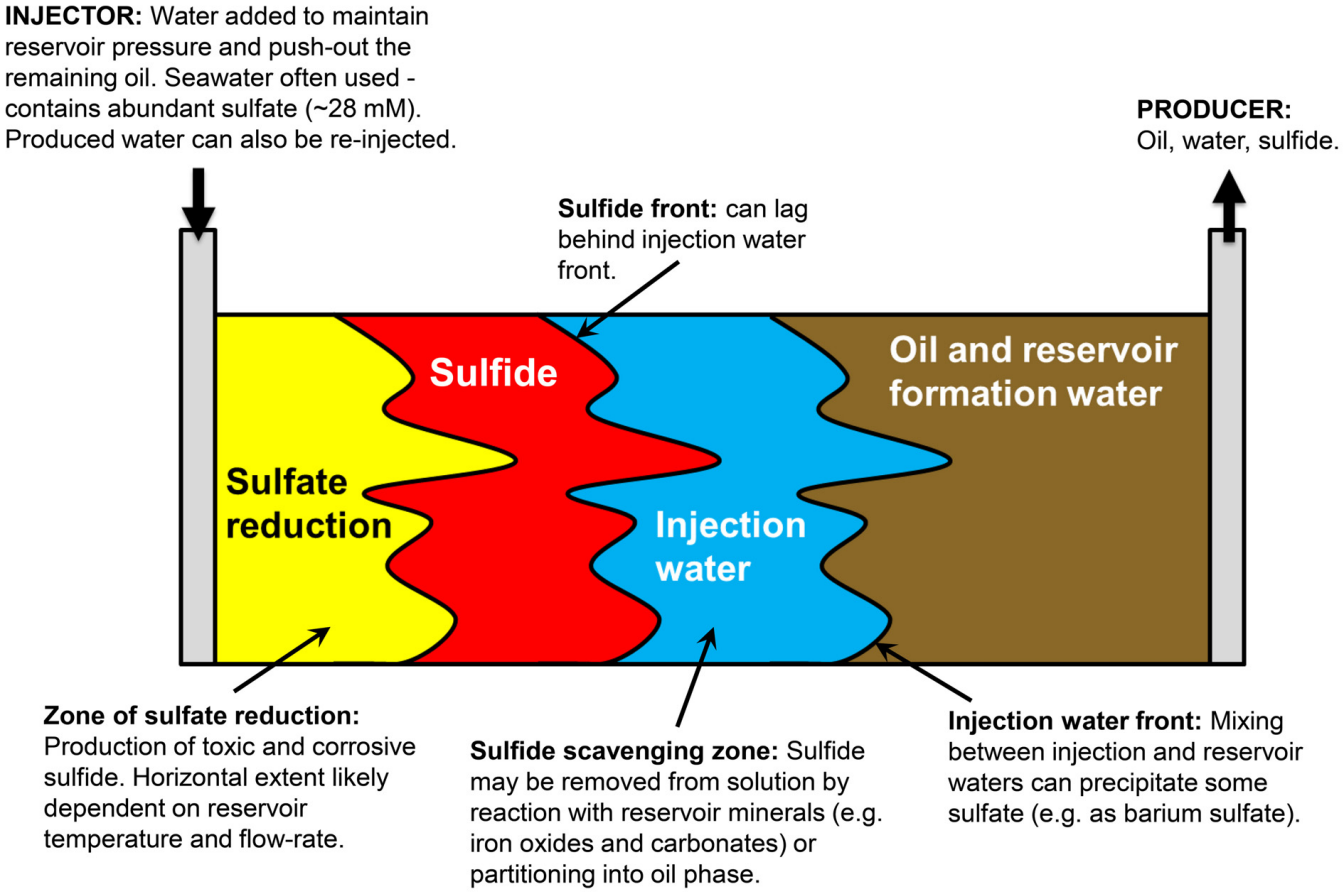
**Conceptual model of reservoir souring processes (after http://www.oilfieldwiki.com/wiki/Reservoir_souring)**.

Figure 1 presents a conceptual model of microbial souring. During secondary production of oil, water is injected into the oil reservoir to provide reservoir pressure and to sweep oil toward production wells. In offshore environments, the injection water (IW) is usually seawater, which contains abundant sulfate (~28 mM) as an available electron acceptor. This high sulfate water mixes with any reservoir formation water (FW) present, which often contains elevated concentrations of potential electron donors in the form of volatile fatty acids (VFAs), such as propionate, butyrate and acetate (Warren et al., 1994; Grigoryan et al., 2008). Sulfate reducing microorganisms (SRM) have also been shown to use more recalcitrant electron donors, such as aliphatic and aromatic hydrocarbons that are components of crude oil (Aeckersberg et al., 1991; Widdel and Bak, 1992; Bolliger et al., 2001; Davidova et al., 2006; Agrawal et al., 2012). The injected water also cools down the reservoir around the injection well to temperatures which are more conducive to microbial sulfate reduction and sulfide production (Eden et al., 1993; Gieg et al., 2011). A zone of optimal sulfate reduction is therefore created, with its location and extent controlled by the thermal and chemical gradients resulting from water injection, and the distribution and nature of the SRM that are present. Transport of sulfide through the reservoir toward the production well can be delayed with respect to the IW front due to the initial lag and growth phase of sulfate reducers, and also due to reaction of sulfide with iron-bearing reservoir minerals, removing the sulfide from solution and precipitating it as iron sulfide (Eden et al., 1993; Coombe et al., 2010). However, the residual sulfate that has not been reduced to sulfide will remain in solution and be transported with the water flood—monitoring changes in the isotopic composition of this sulfate in the produced water (PW) from the reservoir is the key to the concept of isotopes as early indicators of microbial souring. Finally, if the FW contains elevated concentrations of Ba, Sr, or Ca, sulfate may be removed abiotically as insoluble minerals, resulting in mineral scale accumulation that can cause formation damage by decreasing local permeability (Moghadasi et al., 2006; Merdhah and Yassin, 2007). Isotopes can potentially be used along with fluid chemistry data to distinguish between these different abiotic and biotic mechanisms affecting dissolved sulfate concentrations.

In terms of isotope geochemistry, microbial sulfate reduction is one of the most highly fractionating processes known. Microbes prefer to reduce sulfate containing the lighter ^32^S isotope rather than the heavier ^34^S, because ^32^S forms slightly weaker bonds that are easier to break. This produces sulfide that is lighter than the parent sulfate (e.g., Kaplan and Rittenberg, 1964; Bolliger et al., 2001). Over time, the sulfate that remains becomes isotopically heavier as the lighter ^32^S is preferentially consumed. Isotopic abundance is conventionally represented by the delta notation in equation (1), whereas isotope fractionation can be most simply described by the Rayleigh fractionation outlined in equation (2). Experiments have shown that the isotope fractionation factor (ε) in equation (2) can vary substantially between +5 and −66‰, and is thought to depend on a wide variety of factors including electron donor type, electron donor/acceptor concentration, sulfate reduction rate, temperature and the microbial population that is present (Brüchert, 2004; Mitchell et al., 2009; Sim et al., 2011a; and references therein). Mechanistically, these factors may control the relative rates of the reversible enzymatic pathways within the cell itself that are responsible for the isotope fractionation (Brunner and Bernasconi, 2005; Brunner et al., 2005). Understanding the controls on and values of typical isotopic fractionation factors for oil reservoirs is clearly important in order to utilize isotopes as a fully quantitative tool for microbial souring.

Sulfur isotope ratios are commonly reported in standard delta notation, δ^34^S (units of per mil, ‰), where *R*_sample_ = (^34^S/^32^S)_sample_ and *R*_std_ = (^34^S/^32^S)_std_. *R*_std_ is the Canyon Diablo Troilite standard (=0.0441626):

(1) $$\delta^{34}\text{S}=\left[\left(\frac{{\text{R}}_{\text{sample}}}{{\text{R}}_{\text{std}}}\right)-1\times 1000\right]$$

Sulfur isotope fractionation can be described by the simple Rayleigh fractionation in equation (2) where the δ^34^S of the reactant (e.g., sulfate in microbial sulfate reduction) is dependent on the isotope fractionation factor (ε), the fraction of initial reactant remaining (f) and the δ^34^S of the initial reactant. Note that while this equation is often sufficient to describe simple batch experiments (see Sections Experimental and Batch experiments), our modeling approach outlined in Section Representation of sulfur isotope fractionation kinetics. uses a more sophisticated numerical treatment based on a modification of the Monod rate law.

(2) $$\delta^{34}\text{S}=\varepsilon \text{lnf}+\delta^{34}{\text{S}}_{\text{initial}}$$

To date, field applications of isotopes to the souring problem have largely been restricted to using sulfur isotopes to differentiate between H_2_S formed from highly fractionating microbial sulfate reduction and H_2_S from abiotic thermochemical sulfate reduction (Aplin and Coleman, 1995; Poli et al., 2002; Cavallaro et al., 2005; Martins and Marques, 2006). This is based on the observation that thermochemical sulfate reduction often shows an apparent zero fractionation between reservoir minerals and H_2_S, thought to result from when the sulfate reduction itself is kinetically faster than the release of sulfate to solution (Machel et al., 1995). While this is undeniably useful in defining whether or not interventions targeting microbial sulfate reduction will be helpful, there is scope for greater use of isotopes in the context of biosouring (Aplin and Coleman, 1995; Carrigan et al., 1997; Hubert et al., 2009). We present the results of simple batch experiments combined with reactive transport modeling to show how sulfate isotopes can potentially be used as early indicators of microbial souring, and to help distinguish between biotic and abiotic processes affecting the concentrations of dissolved sulfate (Figure 1). Reservoir modeling is a key tool used by the oil industry to understand and predict oil production, so integrating isotopic and biological processes (as well as physical and chemical processes) into reservoir models is essential to transitioning our evolving understanding into a product of practical use by industry. These model simulations represent a first order, simplified investigation of a complex, multiphase system, so we also consider how differences in reservoir thermal, geochemical, hydrological, operational, and microbiological conditions may interact to affect microbial souring dynamics and hence the anticipated isotopic signatures.

## Materials and methods

### Experimental

In order to obtain isotope fractionation factors to use in our model simulations, we performed two sets of simple batch experiments. In the first set, microbial sulfate reduction was investigated using crude oil as the carbon source. Approximately 25 mL of water saturated San Francisco Bay sediment was added in duplicate to 150 mL serum bottles as the microbial inoculum, along with 50 mL of San Francisco Bay water as the sulfate source. The bottles were N_2_-purged, crimp sealed, and 1 mL of crude oil was added to each as the electron donor. The bottles were incubated at 30°C and samples were taken periodically over a 125 day period. Sulfate was analyzed by ion chromatography and dissolved sulfide was analyzed spectrophotometrically using a version of the Cline (1969) assay modified for use in a multiwell plate reader. For isotopic analyses, dissolved sulfide was first precipitated from filtered samples by the addition of excess zinc acetate. The zinc sulfide formed was then purified by rinsing with ammonium hydroxide followed by three rinses of deionized water. After removing the sulfide, the remaining liquid was refiltered and sulfate was precipitated as barite (BaSO_4_) by acidifying the sample with hydrochloric acid and adding excess barium chloride. The barite formed was rinsed with deionized water. All samples were dried prior to analysis. Isotope ratios were measured using a Eurovector model 3028 elemental analyzer in helium continuous flow mode interfaced with a GV Isoprime isotope ratio mass spectrometer. The 1σ reproducibility for δ^34^S was ± 0.17‰.

The second set of experiments was focused on barite precipitation. Sodium sulfate solutions (0.1 M) were acidified with hydrochloric acid and titrated with 0.5 M barium chloride at room temperature (~22°C) to precipitate different fractions of the initial sulfate as barite. After precipitation, the solution was filtered and the remaining sulfate was also precipitated as barite. The samples were rinsed, dried, and analyzed for δ^34^S as described previously.

### Modeling

A reactive transport simulator, CrunchTope, was used to systematically elucidate the impacts of biotic processes and abiotic mechanisms (e.g., mineral precipitation and physical mixing) on sulfate and sulfide concentrations and sulfate isotopes. CrunchTope is an extension of CrunchFlow (Steefel and Maher, 2009), a multicomponent model that simulates biogeochemical and transport processes (Li et al., 2010, 2011; Surasani et al., 2013). CrunchTope extends the capabilities of CrunchFlow to include explicit representation of the kinetics of the individual isotopoloques of the chemical species under investigation (Druhan et al., 2012, 2013, 2014). This extension will be discussed in more details in the following subsections.

#### Model setup and parameters

Simulations were conducted under 1D flow conditions to model reactive transport processes between an injection well and a producing well 150 m apart. The simulation domain consisted of 150 nodes, each with a resolution of 1 m. Porosity and permeability were set at 0.2 and 1.0 × 10^−10^ m^2^ respectively. A constant pressure gradient was maintained such that flow velocity was 0.288 m/day, a value within the range of flow velocities selected in previous oil reservoir modeling studies (Farhadinia et al., 2010). The dispersion coefficient was set at 0.40 m, a value consistent with previous reactive transport studies in well-studied near-surface aquifers (Li et al., 2009; Druhan et al., 2012).

The initial concentrations of aqueous species matched that of the FW found in sample #158 from Warren et al. (1994). This sample was taken from the Brent sandstone reservoir in the Oseberg Field of the northern province of the North Sea. The carbon source in the simulation was represented simply as acetate, which has been measured at concentrations up to 18 mM in North Sea FWs (Warren et al., 1994). In this study we used a concentration of 10 mmol/kg H_2_O acetate in the FW. For the IW, we assumed an operating scenario of PW reinjection (e.g., Haghshenas et al., 2012), where the IW was a mixture of 75% seawater (SW) and 25% FW, i.e., IW = 0.75 SW + 0.25 FW. An additional source of nitrogen was introduced as 0.6 mmol/kg H_2_O ammonium bisulfite (NH_4_HSO_4_), which is a common chemical used to scavenge oxygen from IWs in order to minimize oxidative corrosion (Kelland, 2009). We have assumed that all the bisulfide is transformed to sulfate by reaction with oxygen and that the isotopic composition of this sulfate is the same as seawater sulfate. For simplicity, the IW was kept constant in each simulation i.e., not adjusted to reflect temporal changes in the PW. Detailed aqueous species concentrations in FW, SW, and IW can be found in Table 1.

**Table 1:** **Aqueous species concentrations in formation water (FW), seawater (SW), and injection water (IW)**.

**Species**	**Formation water (FW) (mmol/kg H_2_O)**	**Seawater (SW) (mmol/kg H_2_O)**	**Injection water (IW) (mmol/kg H_2_O)**
pH	6.0	8.2	6.6
Na(I)	631	486	522
K(I)	7.3	10.6	9.8
Mg(II)	4.8	54.7	42.3
Ca(II)	23.2	10.7	13.8
Ba(II)	0/10	0	0
Fe(II)	0	0	0
NH_4_(I)	0	0	0.6
Cl(-I)	668/688	566	591
SO_4_(-II)	0	29.3	22.5
HCO_3_(-I)	15.1	1.8	5.1
Acetate	10	0	2.5
S(-II)	0	0	0

*Chemical concentrations in FW and IW used as initial and amendment conditions respectively in CrunchTope simulations. Barium and chloride concentrations in FW were higher for the simulations examining barite precipitation (see text). FW composition from Warren et al. (1994). SW composition from Millero et al. (2008). IW = 0.75 SW + 0.25 FW + 0.6 mmol/kg_H_2_O NH_4_HSO_3_ (note NH_4_HSO_3_ added as oxygen scavenger and assumed to oxidize to sulfate)*.

#### Representation of microbe-mediated reaction kinetics

Various methods have been developed to quantitatively describe the relationship between microbial growth and energy released during reduction-oxidation reactions. The conceptual approach adopted in CrunchTope relates bacterial growth and energetics by following the method in Rittmann and McCarty (2001). In this framework, SRM (and represented as C_5_H_7_O_2_N) mediate the reaction between an electron donor (acetate in this case) and an electron acceptor (sulfate in this case) to derive energy for growth and maintenance. A dual Monod equation is utilized to mathematically represent the coupled microbial sulfate reduction and acetate oxidation:

(3) $$r=\mu [\text{SRM}]\frac{\left[\text{eDonor}\right]}{[\text{eDonor}]+K_{\text{eDonor}}}\frac{\left[\text{eAcceptor}\right]}{[\text{eAcceptor}]+K_{\text{eAcceptor}}}$$

where *r* (mol/kg H_2_O/day) is the growth rate of the SRM, μ (mol/mol-C_5_H_7_O_2_N/day) is the maximum specific growth rate and *K* (mol/kg H_2_O) is the half saturation (affinity constant) of the electron donor/acceptor. The terminal electron accepting process, sulfate reduction, consists of two components: catabolic and anabolic. This means for each mole of electron donor/substrate utilized, a portion, *fs*, is conserved by the SRM for cell synthesis (anabolic) while the remaining fraction, *fe*, is used for energy production (catabolic) (Rittmann and McCarty, 2001). For all simulations in this study, we assume *fs* = 0.08 and *fe* = 0.92, in agreement with previous reactive transport modeling studies that simulate SRM metabolism (Fang et al., 2009; Druhan et al., 2012, 2014). Specifically, 8% of the electrons are utilized for cell synthesis while the remaining 92% are used for energy production. For each time step, the volume fraction of SRM is updated as:

(4) $$B^{t+\Delta t}=B^{t}+rM\Delta t$$

where M is the molar volume of cells (m^3^cells/mole). In CrunchTope, the SRM population is assumed to be dominantly sessile. This assumption is consistent with literature observations that microbial cells tend to form biofilms in natural subsurface environments (Rittmann and McCarty, 2001). The decay of biomass is modeled with a first order decay model with a decay constant of 0.00027/day following Druhan et al. (2012).

#### Representation of sulfur isotope fractionation kinetics

Druhan et al. (2012, 2014) extended the capabilities of CrunchFlow to include explicit representations of the kinetics of the individual isotopologues of sulfur, ^32^*SO*^2−^_4_ and ^34^*SO*^2−^_4_ (Druhan et al., 2012, 2014) through the modification of the Monod rate law.

(5) 32r= 32μ[SRM][S32O42−][S32O42−]+32Ks (1 + [S34O42−]K34s)

(6) 34r= 34μ[SRM][34SO42−][34SO42−]+34Ks (1+[S32O42−]32Ks )

By assuming a common half saturation constant for both ^32^*r* and ^34^*r*, Druhan et al. (2012) derived the following dual Monod rate laws that also incorporate a dependency on electron donor (acetate) concentration:

(7) $${}^{32}r={\;}^{32}\mu \left[SRM\right]\frac{\left[{}^{32}SO_{4}^{2-}\right]}{\left[SO_{4}^{2-}\right]+K_{S}^{SO_{4}}}\frac{\left[Ac\right]}{[Ac]+K_{S}^{Ac}}$$

(8) $${}^{34}r={\;}^{34}\mu \left[SRM\right]\frac{\left[{}^{34}SO_{4}^{2-}\right]}{\left[SO_{4}^{2-}\right]+K_{S}^{SO_{4}}}\frac{\left[Ac\right]}{[Ac]+K_{S}^{Ac}}$$

As a result, the fractionation factor (α) can be calculated as shown below. Note that the fractionation factors α and ε are related according to ε ≈ 1000.(α − 1).

(9) $$\alpha =\frac{{}^{34}\mu }{{}^{32}\mu }$$

#### Representation of mineral kinetics

Rates of mineral dissolution and precipitation are calculated based on a rate law derived from the Transition State Theory (TST) as postulated by Lasaga (1998).

(10) $$r=Ak\left(1-\frac{IAP}{Keq}\right)$$

Where *k* is the reaction rate constant, *IAP* is the ion activity product, *K*_*eq*_ is the equilibrium constant of the reaction and *A* is the reactive surface area of the mineral.

#### Model simulations

The following simulations were designed to address the previously posed science questions. Specifically, to systematically demonstrate the impacts of iron minerals and barite precipitation on the evolution of sulfate and sulfide concentrations in PW, and to highlight how changes in the isotopic composition of PW sulfate can be an early indicator of microbial souring.

***Baseline***. A baseline simulation was conducted to simulate the spatio-temporal evolution of the chemical species from the start of water injection to complete IW breakthrough. Microbial sulfate reduction was deactivated in the baseline simulation. The goal of this simulation is to provide a reference for all other simulations.

***Impact of isotope fractionation factor***. As discussed in the introduction, microbial sulfate reduction is one of the most highly fractionating processes known. The isotope fractionation factor (ε) in equation (2) can depend on a wide variety of factors including the type of electron donor, electron donor/acceptor concentrations, sulfate reduction rate, and the microbial population (Brüchert, 2004; Sim et al., 2011a). Understanding the controls on the values of typical isotopic fractionation factors for oil reservoirs is important for using isotopes as a quantitative tool for microbial souring. In order to elucidate the impact of isotope fractionation factor on δ^34^S-sulfate breakthrough, we conducted simulations of microbial sulfate reduction with varying magnitudes of fractionation factor (i.e., ε = −10, −30, −50‰). Note that the −30‰ fractionation factor was taken from our batch experiment results (see Section Batch experiments).

***Impact of iron minerals***. In order to better understand the impacts of iron minerals on delaying the H_2_S breakthrough, we conducted simulations of reservoir sulfate reduction with varying initial amounts of iron mineral (i.e., 0, 0.001, and 0.005% volume fraction). For the purpose of this study we represented the iron mineral phase as Fe(OH)_3_, with the reaction network used in previous reactive transport studies (Fang et al., 2009; Li et al., 2009, 2010; Druhan et al., 2012) and outlined in Table 2. The reaction kinetics of reaction (iii) were half-order with respect to sulfide concentration (Poulton et al., 2004; Dale et al., 2008), and the surface area of the iron hydroxide (2.5 m^2^/g) was consistent with hematite (Poulton et al., 2004). The log*K* of the reaction was within the range of previously used values (Li et al., 2009; Druhan et al., 2012).

**Table 2:** **Reactions modeled and associated kinetic and thermodynamic parameters**.

**Microbe-mediated redox reactions**	**μ (mol/mol-C_5_H_7_O_2_N/day)**	***K*_SO4_**	***K*_AC_**
		**(mol/kg H_2_O)**	
(i) SO^2−^_4_ + 1.082CH_3_CHOO^−^ + 3.05NH^+^_4_ → 0.033C_5_H_7_O_2_N_*SRB*_ + 2.1H_2_O + 2CO_2(*aq*)_ + H_2_S_(*aq*)_	6.85	5.0 × 10^−3^	1.0 × 10^−4^
**Mineral dissolution and precipitation**	**log*k* (mol/m^2^/s)**	**log*K*_eq_**	
(ii) Fe^2+^ + H_2_S_(*aq*)_ ↔ FeS_(am)_ + H^+^	−7.0	3.5	
(iii) Fe(OH)_3(*s*)_ + 0.5H_2_S_(*aq*)_ + 2.5H^+^ ↔ Fe^2+^ + 0.5S_(*s*)_ + H_2_O + 2.0OH^−^	−4.0	−19.6	
(iv) Ba^2+^ + SO^2−^_4_ ↔ BaSO_4(*s*)_	−8.0	−9.97	

*(i) Dale et al., 2008; Fang et al., 2009; Li et al., 2009, 2010; Druhan et al., 2012, 2013*.

*(ii) Li et al., 2009, 2010; Druhan et al., 2012, 2013*.

*(iii) Li et al., 2009, 2010; Druhan et al., 2012*.

*(iv) Windt et al., 2008*.

***Impact of barite precipitation***. In order to better understand the impacts of insoluble sulfate mineral precipitation on sulfate isotopes, we conducted simulations with 10 mmol/kg H_2_O of barium in the FW with and without microbial sulfate reduction. This concentration is within the range published for the North Sea (up to 18 mM; Warren et al., 1994). The barite surface area (0.0107 m^2^/g) was taken from Christy and Putnis (1993) and the precipitation kinetics are described by reaction (iv) in Table 2 (Windt et al., 2008). The fractionation factors used for barite precipitation (ε = +0.4‰) and microbial sulfate reduction (ε = −30‰) were taken from our batch experimental results (Section Batch experiments).

***Impact of physical mixing***. In the previous simulations, the FW contained no sulfate, allowing the examination of the effects of microbial sulfate reduction and sulfate mineral precipitation on the PW sulfate isotopes during water injection. However, conservative mixing between injection and FWs can also play an important role when the FWs do contain appreciable dissolved sulfate, with a different δ^34^S value to the IW. This can be modeled within a reactive transport simulator (such as CrunchTope) but is more clearly illustrated using a simple two-component mixing relationship:

(11) $$\begin{array}{l} \delta^{34}{\text{S}}_{\text{PW}}=(\text{x}.{\text{SO}}_{4-\text{IW}}\delta^{34}{\text{S}}_{\text{IW}}\\ \;+\left(\text{1}-\text{x}\right)\text{​}.{\text{SO}}_{4-\text{FW}}\delta^{34}{\text{S}}_{\text{FW}})/{\text{SO}}_{4-\text{PW}} \end{array}$$

where x = fraction of IW in PW, FW = FW. To explore this parameter space, we have varied the FW sulfate concentrations and δ^34^S relative to the IW, assuming that the IW is seawater (sulfate = 28 mM; δ^34^S = 21‰).

## Results

### Batch experiments

**Figure 2:**
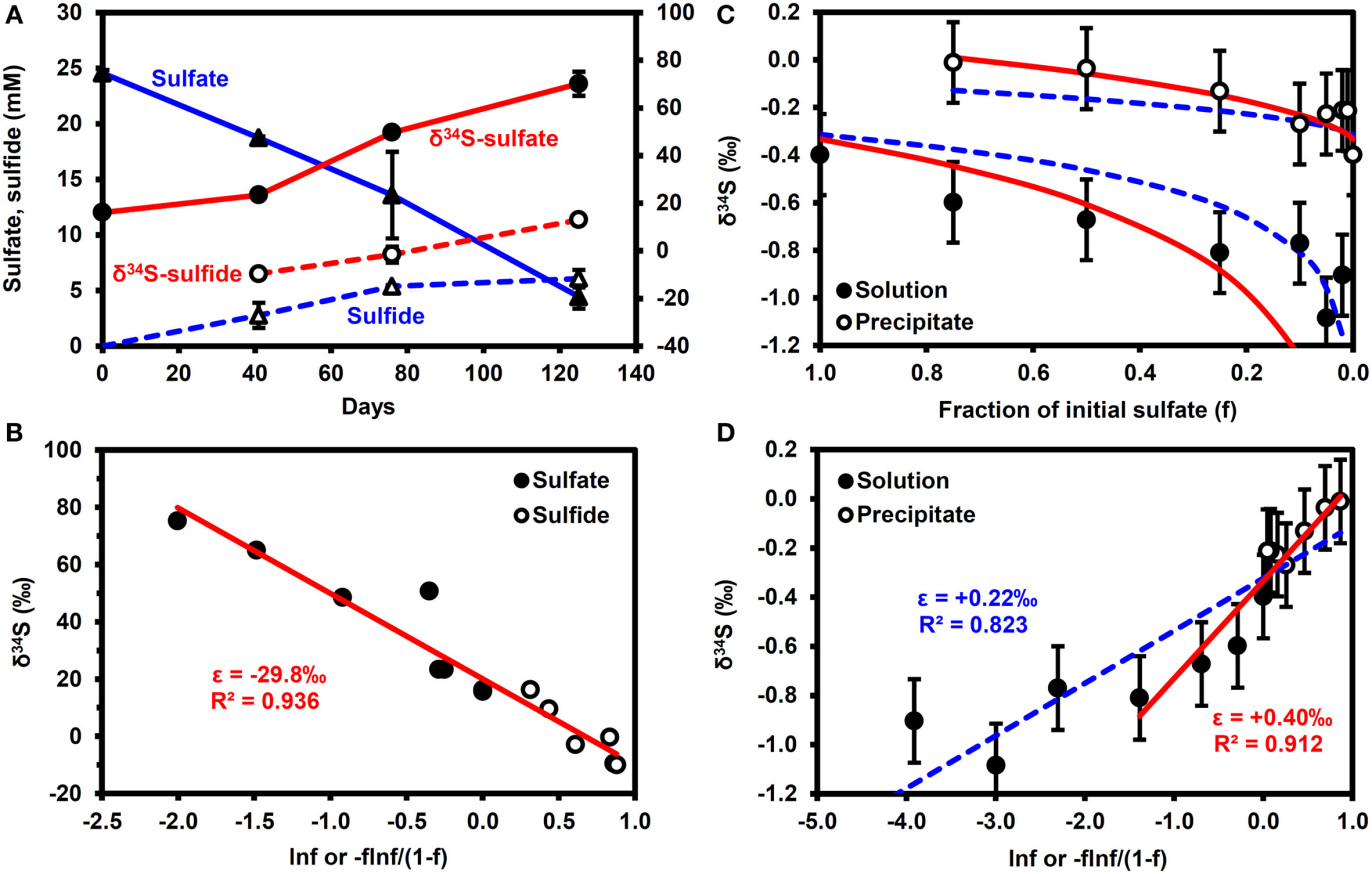
**Results of batch experiments**. **(A,B)** Microbial sulfate reduction experiments performed using San Francisco Bay water as the sulfate source, Bay water/mud as the microbial inoculum and crude oil as the electron donor. Error bars are 1σ of duplicates. **(A)** Time series. **(B)** Rayleigh fractionation. **(C,D)** δ^34^S enrichment in precipitated barite. Error bars are typical 1σ reproducibility of the analytical technique. Solid (red) lines correspond to ε = +0.22‰, dashed (blue) lines correspond to ε = +0.40‰. f, fraction of initial sulfate remaining.

Figure 2A shows the results of the microbial sulfate reduction experiment. Over 125 days, sulfate decreased from 24.6 to 4.4 mM, while dissolved sulfide increased to 6.1 mM. The lack of mass balance in the dissolved sulfur species is likely due to the reaction of sulfide with iron minerals in the bay mud to form FeS_(am)_ or elemental S, as illustrated through reactions (ii) and (iii) in Table 2, as well as partitioning of some H_2_S into the headspace of the serum bottle and into the oil phase. Figure 2B highlights that the shift in δ^34^S values of the sulfate and sulfide conforms to a simple Rayleigh fractionation model, equation (2), with an isotopic fractionation factor (ε) of −29.8 ± 0.05‰ (±1σ). This is similar to the results of Brüchert (2004), who reports fractionation factors of −27 and −36‰ for batch experiments with decane and crude oil, respectively.

Figures 2C,D show that barite precipitation results in a much smaller isotope fractionation effect than microbial sulfate reduction. A fractionation factor of +0.22 ± 0.03‰ (±1σ) can be calculated from the whole dataset, although a fractionation of +0.40 ± 0.08‰ (±1σ) provides a better fit to the data when *f* > 0.2 (*f* = fraction of initial sulfate remaining). Note that precipitation results in a decrease in the δ^34^S of the residual sulfate i.e., a shift in the opposite direction to microbial sulfate reduction.

### Model simulations

#### Impact of microbial sulfate reduction and isotope fractionation factors

**Figure 3:**
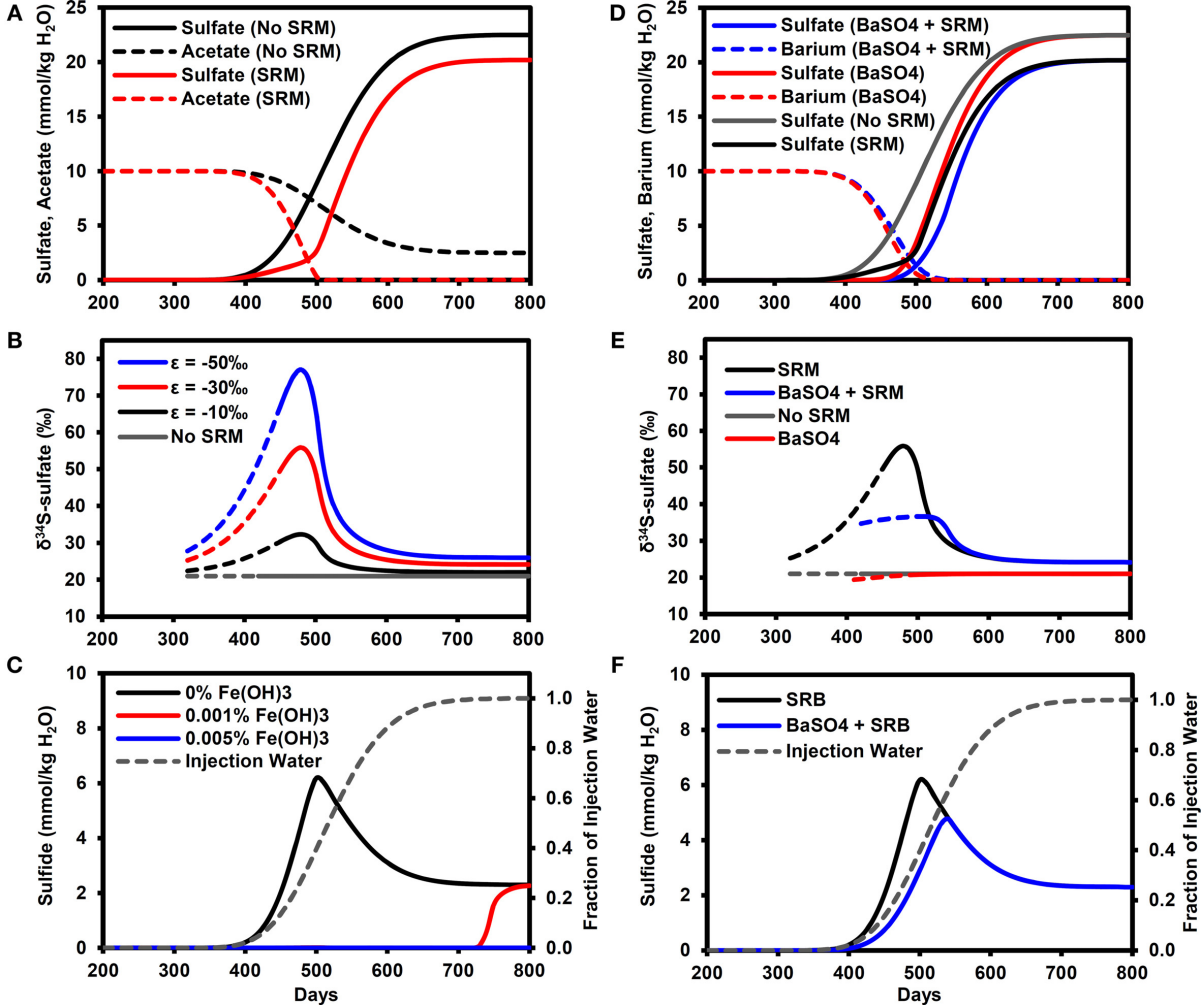
**Breakthrough curves at production well for model outputs**. **(A)** Sulfate and acetate breakthrough with and without microbial sulfate reduction. **(B)** δ^34^S-sulfate breakthrough with and with microbial sulfate reduction (ε = −10, −30, −50‰). Solid lines for sulfate >1 mmol/kg H_2_O (dashed lines for sulfate >1 μmol/kg H_2_O). **(C)** Sulfide breakthrough for different reactive iron mineral content shown with fraction of injection water calculated from chloride data. **(D)** Sulfate breakthrough for different FW Ba concentrations (0, 10 mmol/kg H_2_O) with and without microbial sulfate reduction. **(E)** δ^34^S-sulfate breakthrough with and without barite precipitation (ε = 0.4‰) and microbial sulfate reduction (ε = −30‰). Solid lines for sulfate >1 mmol/kg H_2_O (dashed lines for sulfate >1 μmol/kg H_2_O). **(F)** Sulfide breakthrough shown with fraction of injection water calculated from chloride data. SRM, Sulfate reducing microorganisms.

Figure 3A illustrates the breakthrough of sulfate in the model simulations, with and without microbial sulfate reduction. In the baseline simulation without sulfate reduction, sulfate essentially acts as a conservative tracer of the water flood (compare with Figure 3C) with the PW containing 1% of IW (0.225 mmol/kg H_2_O sulfate) on day 384 (calculated by linear interpolation between time-points), rising to 50% (11.25 mmol/kg H_2_O sulfate) on day 527 and exceeding 99% (>22.3 mmol/kg H_2_O sulfate) on day 690. With microbial sulfate reduction, the sulfate breakthrough curve is delayed with respect to the baseline simulation, reaching 11.25 mmol/kg H_2_O on day 551 with a maximum sulfate concentration of only 20.2 mmol/kg H_2_O. Acetate concentrations in the PW are reduced by SRMs to values equivalent to less than 1% of the FW (i.e., <0.1 mmol/kg H_2_O) by day 510.

The effect of microbial sulfate reduction on the sulfate δ^34^S value is shown in Figure 3B. The solid lines represent sulfate concentrations above 1 mmol/kg H_2_O, at which isotope analyses can be easily performed using the methods outlined earlier in Section Experimental. Values of δ^34^S increase to their maximum on day 480 (PW contains 28% IW), with the exact value depending on the isotopic fractionation factor (δ^34^S = 32.3, 55.9, 77.1‰ for ε = −10, −30, −50‰, respectively). These maximum values correspond to the time when the greatest fraction of initial sulfate was reduced (74%). In these simulations, this maximum was in the mixing front between the formation and IWs, where the sulfate concentrations were diluted by the FW while the acetate concentrations increased (Figure 3A). As sulfate concentrations increased and acetate concentrations decreased with time, the δ^34^S values decreased toward a dynamic steady state representing the consumption of all acetate in the IW, equivalent to consuming ~10% of the injected sulfate. Note that these values (δ^34^S = 22.0, 24.2, 26.0‰ for ε = −10, −30, −50‰, respectively) are all analytically distinguishable from the IW value of 21.0‰.

#### Impact of reservoir iron minerals

For the microbial sulfate reduction simulation with no reservoir iron minerals, H_2_S concentrations increase above 0.01 mmol/kg H_2_O on day 360, reaching a peak of 6.2 mmol/kg H_2_O on day 500 (PW contains 40% IW) before decreasing to 2.3 mmol/kg H_2_O (Figure 3C). Note that the peak production of sulfide also occurred in the mixing front between the formation and IWs. With only 0.001% by volume reactive iron mineral phase, as Fe(OH)_3_, sulfide did not exceed 0.01 mmol/kg H_2_O until day 710 (PW contains almost 100% IW) and rose to 2.3 mmol/kg H_2_O. At the higher volume fraction of 0.005% Fe(OH)_3_, sulfide did not exceed 0.01 mmol/kg H_2_O during the simulation. For all these cases, it is important to bear in mind that the δ^34^S-sulfate signatures shown in Figure 3B will not be affected by the sulfide removal.

#### Impact of barite precipitation

Figure 3D shows the effect of barite precipitation on the sulfate breakthrough curves with and without microbial sulfate reduction. The sulfate breakthrough curve is delayed by barite precipitation. In the simulation without barite precipitation or microbial sulfate reduction, sulfate increased to 11.25 mmol/kg H_2_O on day 527 (same as Figure 3A). In the simulation with only barite precipitation, this concentration was reached on day 543, whereas in the simulation with only microbial sulfate reduction, this concentration was reached on day 551. Finally, in the simulation with both barite precipitation and microbial sulfate reduction, this sulfate concentration was reached on day 568.

Barite precipitation alone has very little effect on the sulfate δ^34^S (Figure 3E), with a δ^34^S value of 20.9‰ on day 490 (when sulfate = 0.9 mmol/kg H_2_O), i.e., close to the seawater sulfate value of 21.0‰. This means that when microbial sulfate reduction also occurred, the δ^34^S-sulfate signature was dominated by the microbial sulfate reduction signature. However, the peak δ^34^S for barite precipitation and microbial sulfate reduction (35.1‰ on day 520) is lower than the peak for microbial sulfate reduction alone (55.9‰ at day 480). Figure 3F illustrates that peak sulfide is also lower (4.5 mmol/kg H_2_O) and delayed (day 540) in comparison to microbial sulfate reduction without barite precipitation (6.2 mmol/kg H_2_O on day 500).

#### Impact of physical mixing

**Figure 4:**
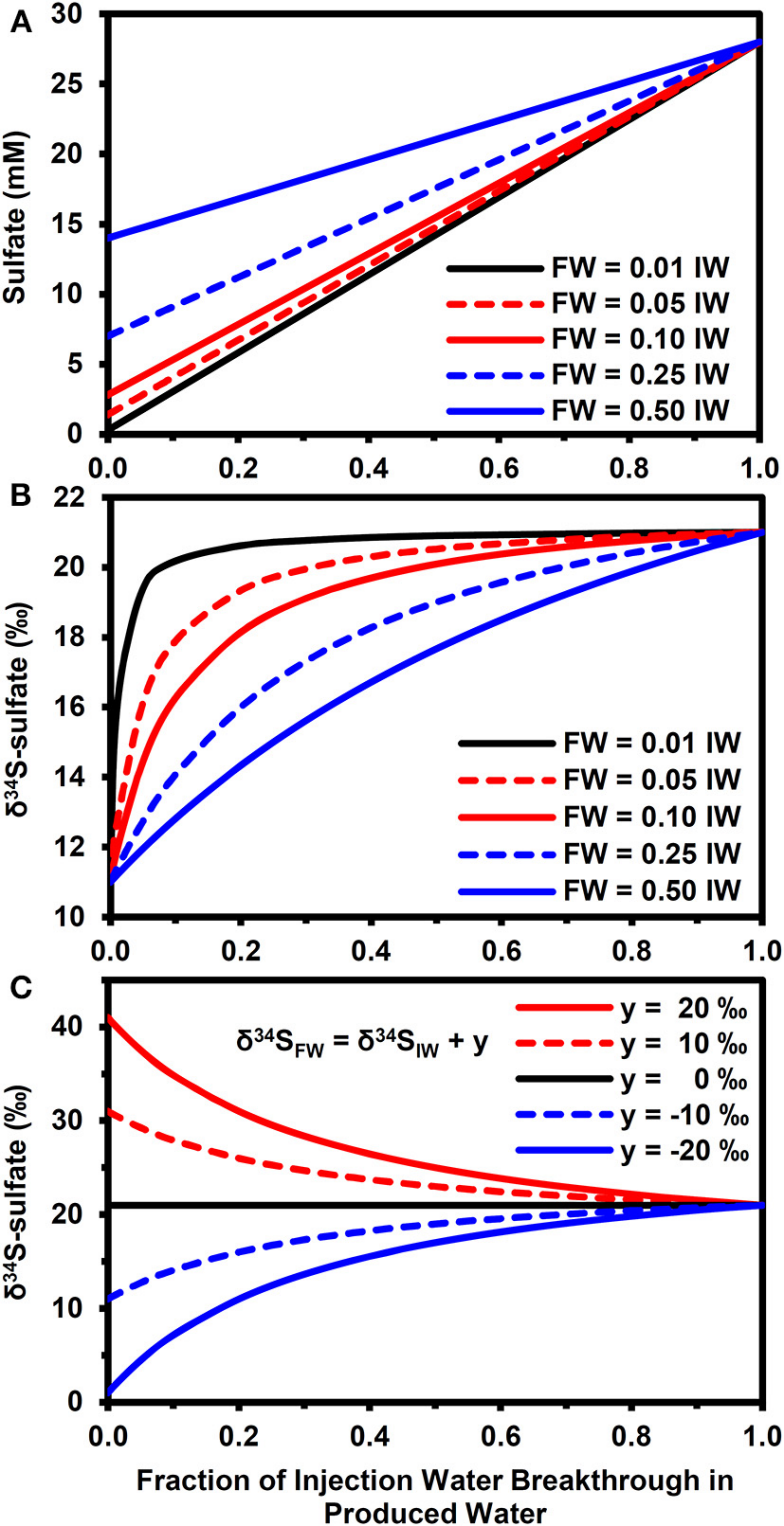
**Effect of physical mixing on breakthrough of injection water**. **(A)** Evolution of sulfate concentrations during injection water breakthrough for different formation water compositions (SO_4−FW_ = *z*.*SO*_4−IW_, where SO_4−IW_ = 28 mM and *z* = 0.01, 0.05, 0.1, 0.2, 0.5). **(B)** Evolution of δ^34^S-sulfate for compositions in **(A)** when δ^34^S_FW_ = δ^34^S_IW_ − 10‰ and δ^34^S_IW_ = 21‰. **(C)** Evolution of δ^34^S-sulfate for fixed FW composition (SO_4−FW_ = 0.25.SO_4−IW_) when δ^34^S_FW_ = δ ^34^S_IW_ + *y*, where *y* = 20, 10, 0, −10, −20‰.

Figure 4 highlights how mixing between injection and FWs can affect the sulfate concentrations (Figure 4A) and δ^34^S-sulfate, according to equation (11). Note that these results are for physical mixing processes only. That is, there was no isotopic fractionation induced by mineral precipitation or microbial sulfate reduction. This approach allows us to explore the parameter space in a more exhaustive and efficient manner than using a full reactive transport simulator. For the scenario with a 10‰ difference between injection and FW δ^34^S-sulfate (Figure 4B), the PW δ^34^S rapidly became dominated by the IW composition during IW breakthrough when the FW sulfate concentration is low relative to IW (e.g., SO_4−FW_ = 0.01.SO_4−IW_). However, it took a longer time to approach the IW composition when FW sulfate was higher (compare with SO_4−FW_ = 0.5.SO_4−IW_, Figure 4B). Larger differences between formation and IW δ^34^S-sulfate also increased the time needed for PW δ^34^S to approach IW values (Figure 4C).

## Discussion

Our modeling results clearly show the potential of stable isotopes as early indicators of microbial souring. The presence of low volume fractions of reactive iron mineral phases was sufficient to delay the breakthrough of sulfide relative to an unambiguous isotopic signature of microbial sulfate reduction shown by the increase in the δ^34^S values of dissolved sulfate, when compared with the IW value. The effect of microbial sulfate reduction on δ^34^S dominates over the comparatively minor isotope fractionation associated with barite precipitation (Figures 2, 3E), although physical mixing does need to be taken into account when FW sulfate concentrations are relatively high and/or there is a large difference between formation and IW δ^34^S (Figure 4). It is also worth considering further how differences in reservoir thermal, geochemical, hydrological, operational and microbiological conditions can interact to affect microbial souring dynamics and hence the anticipated isotopic signatures.

One of the most notable results of the modeling is that the largest shift in δ^34^S occurred in the mixing zone between FW containing elevated electron donor and the IW containing high sulfate (Figures 3A,B). These concentration gradients lead to a scenario whereby a high fraction of the sulfate can be reduced to sulfide, leading to a pronounced increase in the δ^34^S of the residual sulfate. This is a very promising result from a monitoring perspective, suggesting that monitoring during the water flood breakthrough may give the clearest results. However, it should be noted that competition for sulfate between microbial sulfate reducers and mineral scale formation (i.e., Ba/Ca/Sr sulfate precipitation) will affect this, as shown in Figures 3D–F. The relative kinetics between the processes will be important here and may vary according to temperature, chemistry and whether or not scale inhibitors are being used; scale inhibiting chemicals will decrease the nucleation and crystallization rates of mineral scales (He et al., 1994; Shen et al., 2009).

Microbial sulfate reduction rates in the mixing zone will vary depending on (i) concentration and nature of the available electron donors, (ii) thermal regime, (iii) population (number and nature) of the sulfate reducers. Our modeling example is isothermal (25°C) with a relatively high concentration of accessible electron donor (VFAs represented as acetate), providing good starting conditions for growth and metabolism of mesophilic SRMs. However, in an oil reservoir with low concentrations of VFAs, sulfate reduction rates may be slower and may be coupled to the oxidation of more recalcitrant components such as BTEX (e.g., toluene) and short chain aliphatic hydrocarbons (Bolliger et al., 2001; Davidova et al., 2006; Agrawal et al., 2012). The change in thermal regime caused by injecting cold seawater into a relatively hot reservoir will result in an evolving spatial temperature profile that will favor mesophiles, thermophiles, and hyperthermophiles at different distances from the injector. The resulting spatiotemporal profiles in sulfate reduction rate will depend on the initial population and thermal optimum of the indigenous oil reservoir microbial population, together with the growth and establishment of any SRMs introduced by the water injection itself. Ultimately, the souring development and δ^34^S signature will therefore depend on how the microbial dynamics of the system interact with the geochemical and thermal gradients imposed by water injection and mixing with any FW.

In our model we represented reservoir iron mineralogy as a simple ferric hydroxide phase, Fe(OH)_3_, with kinetics and surface area equivalent to hematite, a common cement in sandstones. This system has been extensively studied (e.g., Poulton et al., 2004) but the appropriate iron mineralogy (and hence kinetics) in a specific oil reservoir may be very different and could instead be comprised of carbonates (e.g., siderite, ankerite, or ferroan calcites) or aluminosilicates (e.g., iron bearing clays). Only 0.001% volume fraction of Fe(OH)_3_ was needed in our simulations to delay the sulfide breakthrough by approximately 1 year. However, this assumes that the whole mineral surface area is available for reaction with sulfide. This may not be the case if a significant fraction of the mineral is present as a cement between grains rather than being exposed to solution. The formation of FeS by reaction with sulfide could further reduce available reactive mineral surface by armoring the reactive mineral phase. Finally, preferential flow pathways, especially dual porosity regimes with extensive fracture networks, may limit both the effective mineral surface area available for reaction with sulfide and the contact time. A good understanding of both reservoir mineralogy and flow processes is clearly essential and a combination of batch and flow-through experiments (core floods) will help to constrain appropriate kinetics for modeling and data interpretation.

The results of simple mass balance calculations shown in Figure 4 highlight the importance of conducting baseline surveys to derive the maximum benefit of using stable isotopes as early indicators of microbial souring. To put these results in context, an analysis of 194 FW samples from the North Sea (Warren et al., 1994) shows that 49% of these waters had sulfate concentrations equivalent to less than 1% of seawater sulfate. For these waters, physical mixing is expected to play a minor role in determining PW δ^34^S values when compared with the effect of microbial sulfate reduction (assuming seawater is used as the IW). For FWs with higher sulfate relative to the IW, getting baseline δ^34^S data is of greater importance. In the dataset of Warren et al. (1994), approximately 68% of FWs had sulfate concentrations below 10% of seawater and 94% were below 50% of seawater. Note that stable isotopes of water (i.e., δ^2^H and δ^18^O) have been used successfully as tracers of IW when there are significant differences between injection and FWs (Carrigan et al., 1997; Ahmad et al., 2003) and can therefore be used in conjunction with sulfate isotopes and aqueous concentration data to better constrain the dynamics of water flooding and microbial souring (Carrigan et al., 1997; Huseby et al., 2005). This will enable mixing-induced shifts in δ^34^S (Figure 4) to be differentiated from the impact of microbial sulfate reduction (Figure 2). Some reservoirs may also have baseline values of naturally occurring concentrations of sulfide before water injection has even been started (e.g., Aplin and Coleman, 1995). For these reservoirs, monitoring sulfide concentrations and δ^34^S can potentially be used with sulfate and other aqueous chemistry data to help differentiate between shifts in sulfide concentration due to changing water/gas/oil ratios during production and due to microbial sulfate reduction of the IW (Aplin and Coleman, 1995).

To maximize the usefulness of sulfate isotopes as early indicators of souring, it is important to constrain the values of the isotope fractionation factors (ε) involved. This will help quantify the fraction of sulfate that is being reduced to sulfide and thereby improve predictions of sulfide breakthrough when used in conjunction with reservoir flow models. Figure 3B shows the sensitivity of our simulations to changes in the sulfur fractionation factor for microbial sulfate reduction. Based on our experimental results in Figure 2, we suggest using an isotopic fractionation factor of −30‰ as an initial value for modeling. This is not only similar to previous batch experiments with decane and crude oil (Brüchert, 2004), but it is also within the range of values for batch experiments summarized in Brüchert (2004) and Sim et al. (2011a) for toluene (ε = −18 to −47‰), butyrate (ε = −16 to −36‰), and acetate (ε = −5 to −32‰), all of which have been considered as potential electron donors for microbial souring (Grigoryan et al., 2008; Agrawal et al., 2012). Studies on pure cultures have shown that a decrease in the cell specific sulfate reduction rate (cSRR) can increase the fractionation factor. Sim et al. (2011a,b) showed how a single organism isolated from marine coastal sediments (*Desulfovibrio* sp. strain DMSS-1) could utilize a range of electron donors at cSRRs spanning two orders of magnitude and sulfur isotope fractionation factors varying from −6 to −66‰, while Leavitt et al. (2013) demonstrated how varying the delivery rate of a single electron donor (lactate) for a single organism (*Desulfovibrio vulgaris* Hildenborough) caused a ~50-fold change in sulfate reduction rate and resulted in fractionation factors varying from −11 to −55‰. Temperature variations can also lead to changes in sulfur isotope fractionation. Mitchell et al. (2009) showed a general decrease in the magnitude of ε from −27 to −0.5‰ as temperature increased for two strains of *Archaeoglobus fulgidus*, a hyperthermophilic sulfate reducer that has also been isolated from hot oil field production waters in the North Sea (Beeder et al., 1994). This corresponded to an inverse relationship between cSRR and ε, although they compiled other literature results to note that these trends were not the same in other sulfate reducers. Further work is still needed to investigate how these observed trends of ε with electron donor, temperature and cSRR apply to different oil reservoir communities before they can be incorporated into robust predictive models.

**Figure 5:**
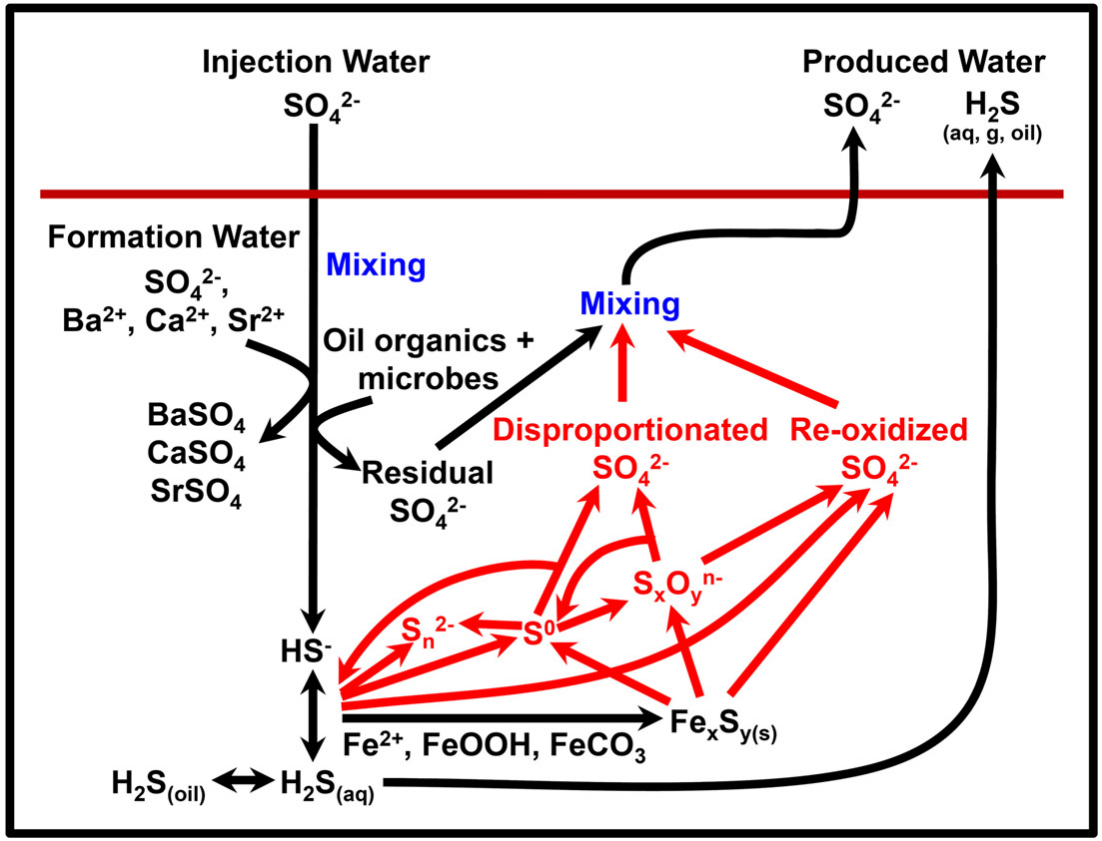
**Potential processes of sulfur cycling and isotope fractionation in oil reservoirs**. Red text and arrows highlight potential additional sulfur cycling due to souring treatments (e.g., nitrate).

So far we have considered a relatively simple network of sulfur cycling. However, the introduction of chemical treatments aimed at preventing souring may, in fact, stimulate a more complex and dynamic sulfur cycle, as illustrated in Figure 5. Nitrate (and nitrite) have been shown to limit sulfidogenesis by multiple mechanism including biocompetitive exclusion of sulfate reducers (by stimulating the thermodynamically preferable process of nitrate reduction), direct inhibition of sulfate reduction by nitrite, and sulfide reoxidation to sulfate by nitrate-reducing sulfide-oxidizers (Jenneman et al., 1986; Nemati et al., 2001; Haghshenas et al., 2012). In comparison, (per)chlorate has more recently been suggested as a souring inhibitor and appears to work by direct inhibition of sulfate reduction and also by stimulating dissimilatory perchlorate reducing bacteria (DPRB) that can oxidize sulfide to elemental sulfur (Ullrich and Huber, 2001; Engelbrektson et al., 2014; Gregoire et al., 2014). The production of sulfur species with intermediate oxidation states (e.g., elemental sulfur or polythionates) leads to the possibility of sulfur disproportionation reactions, although this may depend on whether the oil reservoir has a community with this metabolic capability. Sulfide oxidation to elemental sulfur or to sulfate often leads to small isotope fractionation effects (Toran and Harris, 1989; Hubert et al., 2009; Brabec et al., 2012) whereas disproportionation can result in more significant fractionations (Cypionka et al., 1998; Böttcher et al., 2005). Column studies by Hubert et al. (2009) and Engelbrektson et al. (2014) suggested that nitrate/nitrite treatment can partially mask the effect of sulfate reduction on the sulfate δ^34^S signature, due to reoxidation of isotopically light sulfide accompanied by only a small isotope fractionation. By contrast, oxidation of sulfide to elemental sulfur by DPRB will not affect the sulfate δ^34^S (Engelbrektson et al., 2014), although Liebensteiner et al. (2013) have suggested that a mixed biotic/abiotic pathway by the hyperthermophile *Archaeoglobus fulgidus*, may reoxidize sulfide back to sulfate.

Oxygen isotopes of sulfate provide an additional tool that can be used to investigate both sulfate reduction and the oxidation of reduced sulfur species back to sulfoxyanion species (Böttcher et al., 2005; Hubbard et al., 2009; Brunner et al., 2012). δ^18^O signatures do not follow a Rayleigh fractionation model but rather appear to be dominated by the rapid equilibration of oxygen in the sulfite intermediate species with water-oxygen (Mangalo et al., 2007; Brunner et al., 2012; Müller et al., 2013). As such, this gives us a measurement that is complementary to sulfur isotopes and has the potential for teasing out additional information about dynamic sulfur cycling in oil reservoirs (Hubert et al., 2009).

Overall we have shown the potential for greater use of isotopes in monitoring oil reservoir sulfur cycling. To increase the accuracy of isotope predictions and their use as early indicators of microbial souring, we need to integrate this modeling capability with the type of multiphase reservoir flow models that are used in the oil industry (Coombe et al., 2010; Haghshenas et al., 2012). Further work is also needed to constrain the magnitude of isotope fractionations associated with oil reservoir microbial communities across different temperatures, with varying relevant electron donors, and with the more complex sulfur cycling that could be promoted by treatment chemicals. The ultimate test of this technique will be in applying it to monitoring oil reservoirs undergoing water flood with sulfate-bearing IWs. However, our initial results certainly suggest that further application and development of these isotopic tools will be a worthwhile endeavor.

### Conflict of interest statement

The Associate Editor Dr. Youngblut declares that despite being affiliated to the same institution as the author(s) Dr. Coates, the review process was handled objectively and no conflict of interest exists. The authors declare that the research was conducted in the absence of any commercial or financial relationships that could be construed as a potential conflict of interest.
